# An Extended Chaotic Maps-Based Three-Party Password-Authenticated Key Agreement with User Anonymity

**DOI:** 10.1371/journal.pone.0153870

**Published:** 2016-04-21

**Authors:** Yanrong Lu, Lixiang Li, Hao Zhang, Yixian Yang

**Affiliations:** 1 Information Security Center, State Key Laboratory of Networking and Switching Technology, Beijing University of Posts and Telecommunications, Beijing 100876, China; 2 National Engineering Laboratory for Disaster Backup and Recovery, Beijing University of Posts and Telecommunications, Beijing 100876, China; University of South Australia, AUSTRALIA

## Abstract

User anonymity is one of the key security features of an authenticated key agreement especially for communicating messages via an insecure network. Owing to the better properties and higher performance of chaotic theory, the chaotic maps have been introduced into the security schemes, and hence numerous key agreement schemes have been put forward under chaotic-maps. Recently, Xie et al. released an enhanced scheme under Farash et al.’s scheme and claimed their improvements could withstand the security loopholes pointed out in the scheme of Farash et al., i.e., resistance to the off-line password guessing and user impersonation attacks. Nevertheless, through our careful analysis, the improvements were released by Xie et al. still could not solve the problems troubled in Farash et al‥ Besides, Xie et al.’s improvements failed to achieve the user anonymity and the session key security. With the purpose of eliminating the security risks of the scheme of Xie et al., we design an anonymous password-based three-party authenticated key agreement under chaotic maps. Both the formal analysis and the formal security verification using AVISPA are presented. Also, BAN logic is used to show the correctness of the enhancements. Furthermore, we also demonstrate that the design thwarts most of the common attacks. We also make a comparison between the recent chaotic-maps based schemes and our enhancements in terms of performance.

## 1 Introduction

Authenticated key exchange protocols, are among the core cryptographic mechanisms for ensuring network security, which aims at establishing a common session key between the communicated participates. For authenticated key exchange through an open environment, both security and privacy are desired. Over the past few decades, many works on authenticated key-exchange have been done referring to kinds of cryptographic primitives (e.g., symmetric cryptography, public key cryptography, hash functions, etc.) applied for different applications [1–11].

With infiltration and mergence of many scientific branches, chaotic theory has entered the field of vision of the cryptography researchers. Chaotic theory possesses the properties of unpredictability and sensitivity to parameters and initial conditions, which meet some essential requirements of cryptography. Subsequently, cryptography based on chaos theory has been studied widely. The chaotic maps have been applied in the design of symmetric encryption [12–13], S-boxes [14], signature [15] and hash functions [16]. Additionally, chaotic systems have also been applied to design the key agreements, various chaotic maps-based key agreements and related approaches have been presented recently [17–20], owing to that chaotic maps operations offer the semi-group property, and have a better efficiency than point multiplications on an elliptic curve and modular exponential operations [21–22].

According to the numbers of participants for an authenticated key exchange scheme, there are two-party authenticated key exchange schemes, three-party authenticated key exchange schemes, and multi-party authenticated key exchange schemes. Two-party authenticated key exchange schemes are used to establish a session key under environment of client-server. In particular, the suggestion of three-party authenticated key exchange schemes are considered for solving the infeasibility of two-party schemes exchange session keys in large-scale communication environments. In 2011, Wang et al. [23] developed a three-party authenticated key agreement scheme using chaotic maps. However, Yoon et al. [24] declared that the scheme of Wang et al. violated an illegal message modification attack and then they presented an improvement. Next, Lee et al. [25] presented a chaotic maps based three-party authenticated key agreement scheme without using smart card. However, Hu et al. [26] proved that their scheme was not secure against the man-in-the-middle attack in condition that the identity was lost. After that, Farash et al. [27] proposed a three-party authenticated key agreement without applying symmetric cryptography and server’s public key. Nevertheless, Xie et al. [28] pointed out three-party authenticated key agreement proposed by Farash et al. could not withstand off-line password guessing attack, thus suffering user impersonation attack. In order to prevent the security threats, Xie et al. presented an enhancement without using server’s public key. Obviously, both of Farash et al. and Xie et al.’s schemes are efficient, but without using server’s public key is no guarantee of safety. The most important thing to consider that the identity of user is a key personal privacy. Generally, there is a growing requirement for protecting user privacy information from being leaked and abused, which outlines the needs for designing schemes that can attain user anonymity. The adoption of public key cryptography is essential needed to protect user anonymity, which has been verified by the excellent works [29]. Through our carefully analysis, we found that the proposed scheme by Xie et al. could not achieve user anonymity. In addition, their scheme could not resist off-line password guessing, thus notwithstanding user impersonation attack. Furthermore, the session key security could not provide in their scheme. Motivated by it, we design an extended chaotic maps-based three-party password-authenticated key agreement with user anonymity. Both the formal analysis and the formal security verification using AVISPA [30–31] are presented. Also, BAN logic [32] is used to show the correctness of the enhancements. Furthermore, we also demonstrate that the design thwarts most of the common attacks. We also make a comparison between the recent chaotic-maps based schemes and our enhancements in terms of performance.

The outline of the paper are arranged as follows. The Chebyshev chaotic maps and the related intractable problems are introduced in Section 2. The cryptanalysis of Xie et al.’s scheme is presented in Section 3. Section 4 proposes a chaotic maps-based three-party authenticated key agreement. The security analysis of our scheme and comparison with other works are described in Sections 5 and 6, respectively. We summarize the whole paper in Section 7.

## 2 Preliminaries

We will introduce the Chebyshev chaotic maps and the related intractable problems [33–34].

**Chebyshev polynomial** Let *n* be an integer and *x* ∈ [−1, 1]. The Chebyshev polynomial *T*_*n*_(*x*):[−1, 1] → [−1, 1] can be defined as: *T*_*n*_(*x*) = *cos*(*n* ⋅ *arccos*(*x*)). The recurrent formulas of the Chebyshev polynomial is shown as: *T*_0_(*x*) = 1, *T*_1_(*x*) = *x*, *T*_2_(*x*) = 2*x*^2^ − 1, *T*_*n*+1_(*x*) = 2*xT*_*n*_(*x*) − *T*_*n*−1_(*x*).

**Semi-group property** For $p,q\in N,T_{p}\left(T_{q}\left(x\right)\right)=T_{pq}\left(x\right)=T_{q}\left(T_{p}\left(x\right)\right)\left(modN\right)$.

**Discrete logarithm problem** Known the parameters *x* and *y*, it is intractable to find an integer *p* such that *T*_*p*_(*x*) = *y*.

**Diffie-Hellman problem** Known the parameters *x*, *T*_*p*_(*x*), and *T*_*q*_(*x*), it is intractable to compute the value *T*_*pq*_(*x*).

## 3 Review of Xie et al.’s scheme

**Fig 1:**
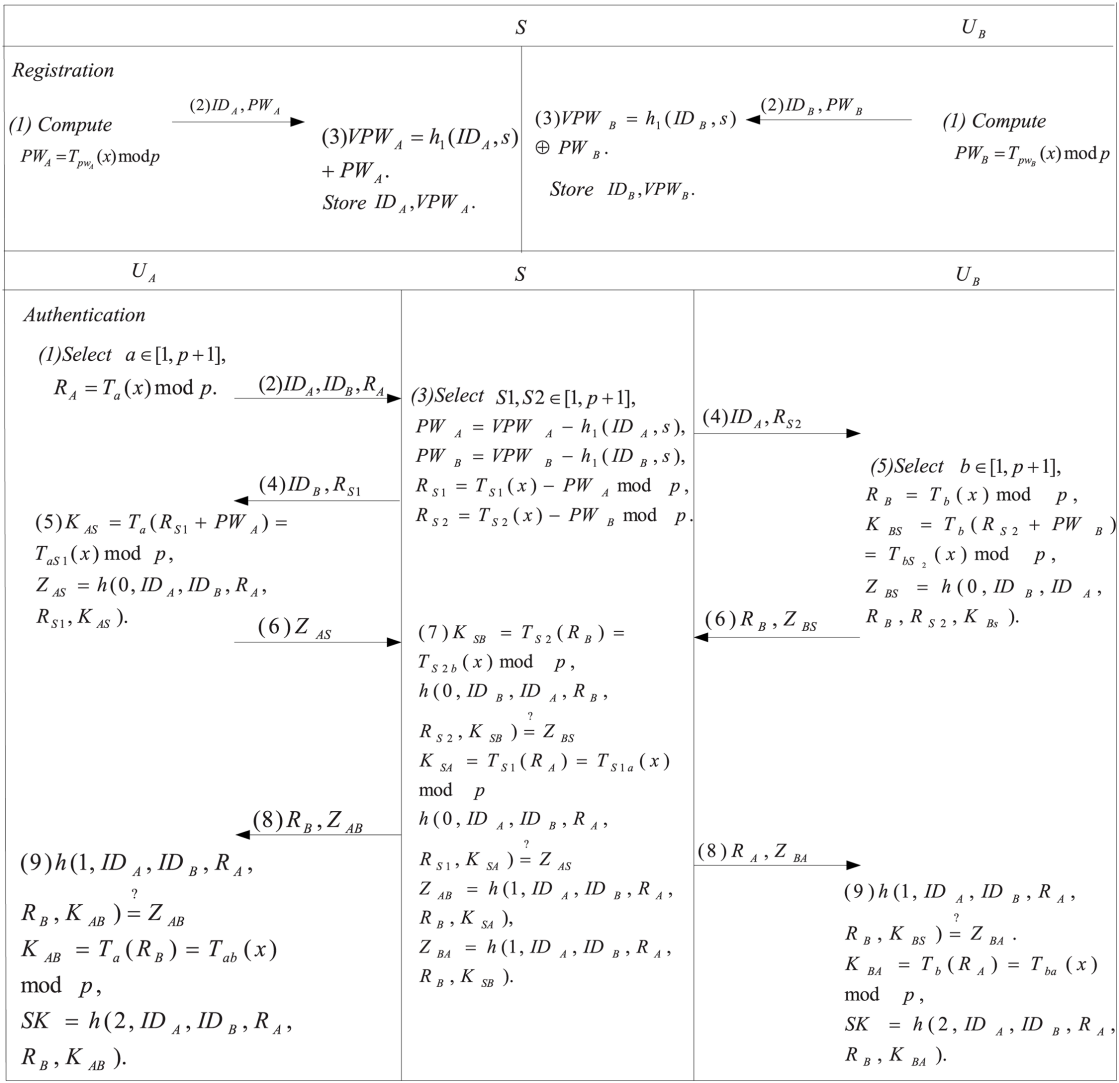
Mutual authentication and key agreement of Xie et al.’s scheme.

In this section, we shall review Xie et al.’s chaotic-maps based authenticated key agreement. Their scheme consists of four phases: system setup, registration, authentication and key exchange and password change. The registration and authentication and key exchange phases are shown in Fig 1. The notations used throughout this study are listed as follows.

*S*: a remote server.

*A* and *B*: two users.

*ID*_*A*_ and *ID*_*B*_: users’ identities of *A* and *B*.

*pw*_*A*_ and *pw*_*B*_: users’s passwords of *A* and *B*.

*k* and *T*_*k*_(*x*): private and public keys of *S*.

*s*: a secret key of *S*.

*r*: shared secret key between *A* and *S*.

*h*_1_(): a one-way hash function *h*_1_: {0, 1}* → {0, 1}^*l*^.

*h*(): a chaotic maps-based one-way hash function $h:{\left\{0,1\right\}}^{*}\to Z_{p}$.

Z: ring of integer.

*p*: a large prime number.

### 3.1 System setup

The server *S* performs the following steps:

Selects its secret key *s*;

Selects a large prime number *p*, $x\in Z_{p}$;

Selects a secure one-way hash function *h*_1_;

Selects a chaotic maps-based one-way hash function *h*().

At last, *S* maintains the secret key *s* and releases the parameters {*p*, *x*, *h*_1_(), *h*()}.

### 3.2 Registration

The user *A* registers the server *S* as below:

Step 1: User *A* computes *PW*_*A*_ = *T*_*pw*_*A*__(*x*)*modp* and sends {*ID*_*A*_, *PW*_*A*_} to *S* through a secure channel, where *ID*_*A*_ and *pw*_*A*_ are the identity and password of *A*, respectively.

Step 2: The server *S* computes *VPW*_*A*_ = *h*_1_(*ID*_*A*_, *s*) + *PW*_*A*_ and stores {*ID*_*A*_, *VPW*_*A*_} in its database.

The user *B* also registers *S* as the above processes, we omit it.

### 3.3 Authentication and key exchange

The establishment of the session key among *A*, *B* and *S* are described in the following:

Step 1: User *A* computes *R*_*A*_ = *T*_*a*_(*x*)*modp* and sends {*ID*_*A*_, *ID*_*B*_, *R*_*A*_} to *S*, where *a* ∈ [1, *p* + 1].

Step 2: Once receiving the login message, *S* computes *PW*_*A*_ = *VPW*_*A*_ − *h*(*ID*_*A*_, *s*), *PW*_*B*_ = *VPW*_*B*_ − *h*(*ID*_*B*_, *s*), *R*_*S*1_ = *T*_*S*1_(*x*) − *PW*_*A*_ *modp*, *R*_*S*2_ = *T*_*S*2_(*x*) − *PW*_*B*_ *modp* and sends back {*ID*_*A*_, *R*_*S*2_} to *B*, sends {*ID*_*B*_, *R*_*S*1_} to *A*.

Step 3: Upon receiving {*ID*_*A*_, *R*_*S*2_} from *S*, *B* computes *R*_*B*_ = *T*_*b*_(*x*)*modp*, *K*_*BS*_ = *T*_*b*_(*R*_*S*2_ + *PW*_*B*_) = *T*_*bS*2_(*x*)*modp*, *Z*_*BS*_ = *h*(0, *ID*_*B*_, *ID*_*A*_, *R*_*B*_, *R*_*S*2_, *K*_*BS*_). Then, *B* sends {*R*_*B*_, *Z*_*BS*_} to *S*. After *A* receives {*ID*_*B*_, *R*_*S*1_} from *S*, he computes *K*_*AS*_ = *T*_*a*_(*R*_*S*1_ + *PW*_*A*_) = *T*_*aS*1_(*x*)*modp*, *Z*_*AS*_ = *h*(0, *ID*_*A*_, *ID*_*B*_, *R*_*A*_, *R*_*S*1_, *K*_*AS*_). Then, *A* sends {*Z*_*AS*_} to *S*.

Step 4: Upon receiving the messages from *A* and *B*, *S* computes *K*_*SB*_ = *T*_*S*2_(*R*_*B*_) = *T*_*S*2*b*_(*x*)*modp* and checks whether $h\left(0,ID_{B},ID_{A},R_{B},R_{S2},K_{SB}\right)\overset{?}{=}Z_{BS}$. If it is true, *S* then computes *K*_*SA*_ = *T*_*S*1_(*R*_*A*_) = *T*_*S*1*a*_(*x*)*modp* and checks whether $h\left(0,ID_{A},ID_{B},R_{A},R_{S1},K_{SA}\right)\overset{?}{=}Z_{AS}$. If holds, *S* computes *Z*_*AB*_ = *h*(1, *ID*_*A*_, *ID*_*B*_, *R*_*A*_, *R*_*B*_, *K*_*SA*_), *Z*_*BA*_ = *h*(1, *ID*_*B*_, *ID*_*A*_, *R*_*B*_, *R*_*A*_, *K*_*SB*_) and sends {*R*_*B*_, *Z*_*AB*_} and {*R*_*A*_, *Z*_*BA*_} to *A* and *B*, respectively.

Step 5: When *A* gets {*R*_*B*_, *Z*_*AB*_}, he verifies whether $h\left(1,ID_{A},ID_{B},R_{A},R_{B},K_{AS}\right)\overset{?}{=}Z_{AB}$. If holds, *A* can compute *K*_*AB*_ = *T*_*a*_(*R*_*B*_) = *T*_*ab*_(*x*)*modp* and the session key *SK* = *h*(2, *ID*_*A*_, *ID*_*B*_, *R*_*A*_, *R*_*B*_, *K*_*AB*_). Similarly, once *B* gets {*R*_*A*_, *Z*_*BA*_}, he verifies whether $h\left(1,ID_{B},ID_{A},R_{B},R_{A},K_{BS}\right)\overset{?}{=}Z_{BA}$. If it is valid, *B* can compute *K*_*BA*_ = *T*_*b*_(*R*_*A*_) = *T*_*ba*_(*x*)*modp* and the session key *SK* = *h*(2, *ID*_*A*_, *ID*_*B*_, *R*_*A*_, *R*_*B*_, *K*_*BA*_).

### 3.4 Password change

If user *A* attempts to update his password as a new one, he can perform the following steps:

Step 1: User *A* computes $PW_{A}^{new}=T_{pw_{A}^{new}}\left(x\right)modp,PWD=h\left(K_{AS},ID_{A}\right)+PW_{A}modp,V_{A}=h\left(K_{AS},PW_{A}\right),Z_{AS}=h\left(1,ID_{A},R_{A},S_{1},K_{AS},V_{A},M_{A}\right)$ and sends {*ID*_*A*_, *R*_*A*_, *Z*_*AS*_, *PWD*, *V*_*A*_, *M*_*A*_} to *S*, where *M*_*A*_ = {Password update request}.

Step 2: *S* first checks whether $h\left(1,ID_{A},R_{A},R_{S1},K_{SA},V_{A},M_{A}\right)\overset{?}{=}Z_{AS}$. If it holds, *S* computes *PW*_*A*_ = *PWD* − *h*(*K*_*SA*_, *ID*_*A*_)*modp* and checks whether $h\left(K_{SA},PW_{A}\right)\overset{?}{=}V_{A}$. If it holds, *S* computes *R*_1_ = *h*(1, *ID*_*A*_, *PWD*, *V*_*A*_, *K*_*SA*_), *VPW*_*A*_ = *h*(*ID*_*A*_, *s*) + *PW*_*A*_ *modp*, replaces *VPW*_*A*_ with $VPW_{A}^{new}$ in its database, and sends {*Accept*, *R*_1_} to *A*. Otherwise, *S* sends {*Reject*, *R*_2_} to *A*, where *R*_2_ = *h*(0, *ID*_*A*_, *PWD*, *V*_*A*_, *K*_*SA*_).

Step 3: When *A* receives {*Accept*, *R*_1_}, he verifies if $h\left(1,ID_{A},PWD,V_{A},K_{AS}\right)\overset{?}{=}R_{1}$. If true, *A* accepts $pw_{A}^{new}$ as his new password. Otherwise, he verifies whether $h\left(0,ID_{A},PWD,V_{A},K_{AS}\right)\overset{?}{=}R_{2}$ and returns Step 1 to execute the above steps again.

## 4 Cryptanalysis of Xie et al.’s scheme

Xie et al.’s scheme declared that their improvements could withstand the password off-line guessing attack and the user impersonation attack which Farash et al.’s scheme failed to resist. However, we will demonstrate their improvement cannot really resist the off-line password guessing attack, thus suffering the user impersonation attack. Besides, we also demonstrate their improvements cannot achieve the session key security as they stated. Furthermore, user anonymity is also not able to provide in their improvements. In order to launch the attacks, we adopt the attack model proposed by Xu et al. [35]. According to their assumption, an attacker U can completely monitor the open communication channel, thus inserting, deleting, and modifying any messages among correspondents.

### 4.1 Off-line password guessing attack

U can easily perform the attack by intercepting the transmitted messages {*ID*_*A*_, *ID*_*B*_, *R*_*A*_} and *Z*_*AS*_ from *A* to *S* as below:

Step 1: U computes *R*_*A*_ = *T*_*a*_(*x*)*modp* and sends {*ID*_*A*_, *ID*_*B*_, *R*_*A*_} to *S*, where *a* ∈ [1, *p* + 1] is a random number.

Step 2: *S* computes *PW*_*A*_ = *VPW*_*A*_ − *h*(*ID*_*A*_, *s*), *PW*_*B*_ = *VPW*_*B*_ − *h*(*ID*_*B*_, *s*), *R*_*S*1_ = *T*_*S*1_(*x*) − *PW*_*A*_ *modp*, *R*_*S*2_ = *T*_*S*2_(*x*) − *PW*_*B*_ *modp*, where *S*1, *S*2 ∈ [1, *p* + 1]. Next, *S* sends {*ID*_*B*_, *R*_*S*1_} to *A*.

Step 3: U guesses a candidate password $PW_{A}^{\prime }$ and computes $K_{AS}=T_{a}\left(R_{S1}+PW_{A}^{\prime }\right)=T_{aS1}\left(x\right)modp$. After that, U checks whether $Z_{AS}\overset{?}{=}h\left(0,ID_{A},ID_{B},R_{A},R_{S1},K_{AS}\right)$. If the equation is true, which means U gets the correct password. Otherwise, U performs the above steps again until he succeeds.

### 4.2 User impersonation attack

After obtaining the password of user *A*(or user *B*), U can masquerade as a legitimate user *A* (or user *B*) to cheat the server *A* and the user *B* (or user *A*). Following previous subsection, once U guesses correctly, he then sends {*Z*_*AS*_} to *S*. Upon receiving the messages from U, *S* executes the original scheme without any detection. Finally, *S* sends {*R*_*B*_, *Z*_*AB*_} to U. After receiving the messages from *S*, U verifies whether *Z*_*AB*_ = *h*(1, *ID*_*A*_, *ID*_*B*_, *R*_*A*_, *R*_*B*_, *K*_*AS*_). If it is true, U computes *K*_*AB*_ = *T*_*a*_(*R*_*B*_) = *T*_*ab*_(*x*)*modp* and the session key *SK*_*AB*_ = *h*(2, *ID*_*A*_, *ID*_*B*_, *R*_*A*_, *R*_*B*_, *K*_*AB*_). That is, U successfully wormed himself into *S* and *B*s’ confidence.

### 4.3 Anonymity of users

The user identity is an important personal privacy. In many cases, U may exploit the user identity to link different login sessions together to trace user activities [29]. Moreover, the violation of user identity and activities may also facilitate an unauthorized entity to trace the user’s login history and even current location [36]. In Xie et al.’s scheme, the messages transmitted from *A* to *S* {*ID*_*A*_, *ID*_*B*_, *R*_*A*_}, sent from *S* to *A* {*ID*_*B*_, *R*_*S*1_}, the message transmitted from *S* to *B* {*ID*_*A*_, *R*_*S*2_}, are all exposed the identity of *A* and *B*. It is a good chance for U to obtain the identity and know who is requiring the service and further trace the position. This means Xie et al.’s scheme fails to achieve user anonymity.

### 4.4 Violation of the session key security

After deriving password *PW*_*A*_ by performing the off-line password guessing attack, U can easily derive the mutually shared session key between *A* and *B* after intercepting the transmitted messages *R*_*A*_ and *R*_*B*_. And thus, U can compute an integer solution a* (or b*) to satisfy the equation $T_{a}^{*}\left(x\right)=T_{a}\left(x\right)$(or $T_{b}^{*}\left(x\right)=T_{b}\left(x\right)$) by adopting the method of Bergamo et al. [22]:

$a^{*}=\frac{arccos(T_{a}\left(x\right))+2k\pi }{arccos(x)}|k\in Z\left(b^{*}=\frac{arccos(T_{b}\left(x\right))+2k\pi }{arccos(x)}|k\in Z\right)$

With the value a* and b*, U can compute the session key: $T_{a}^{*}\left(T_{b}^{*}\left(x\right)\right)modp=T_{a}^{*}\left(T_{b}\left(x\right)\right)modp=T_{b}\left(T_{a}^{*}\left(x\right)\right)modp=T_{b}\left(T_{a}\left(x\right)\right)modp=T_{ba}\left(x\right)modp=K_{AB}$

In this regard, U can compute the session key *SK* = *h*(2, *ID*_*A*_, *ID*_*B*_, *R*_*A*_, *R*_*B*_, *K*_*BA*_) since all the parameters contained in *SK* can be obtained only by intercepting the communication channel.

## 5 Proposed scheme

**Fig 2:**
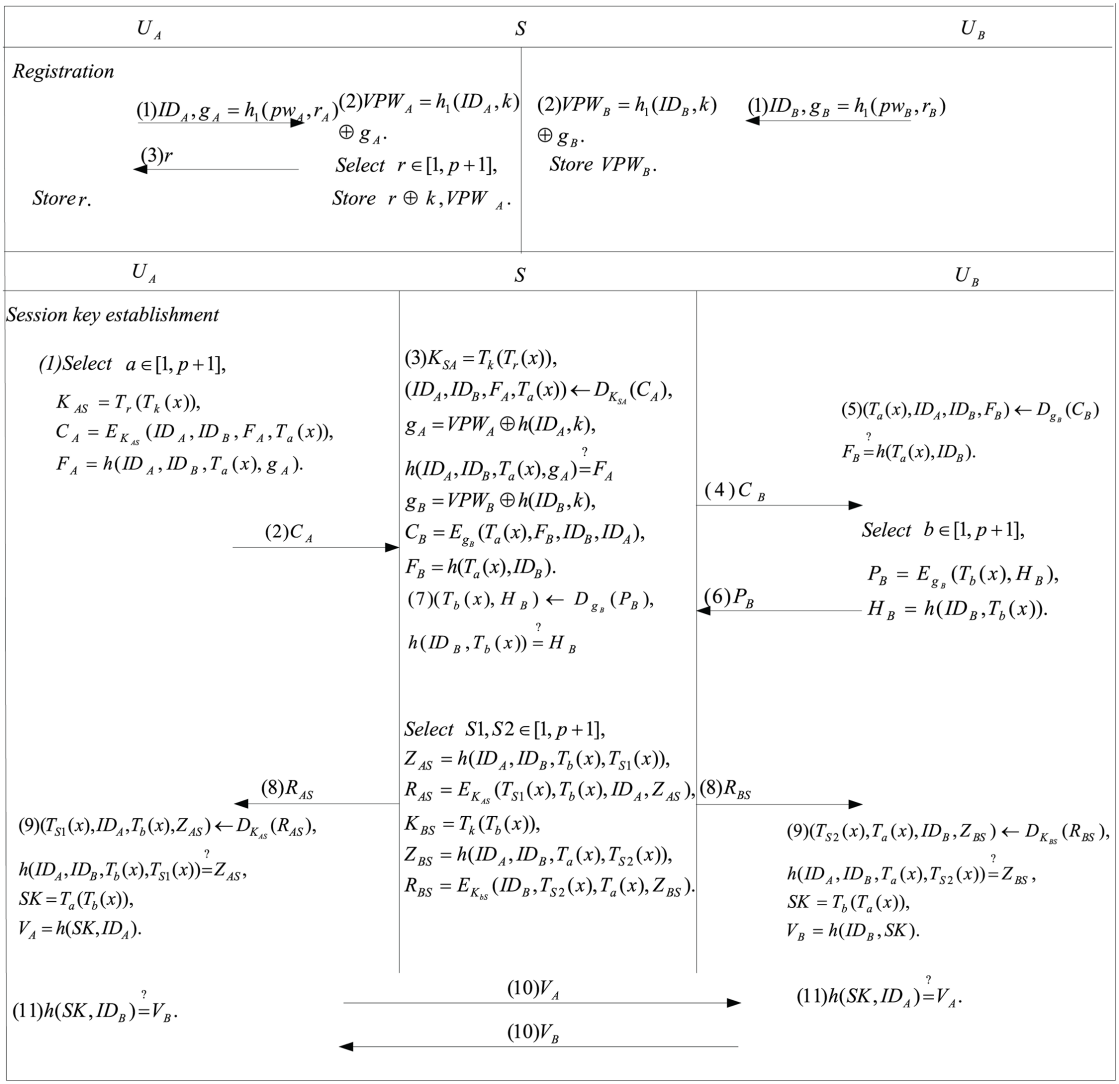
Mutual authentication and key agreement of our scheme.

This section presents our enhanced scheme which inherits the advantages and avoids the disadvantages of the scheme proposed by Xie et al‥ The proposed scheme contains four phases: system initialization, registration, the session key establishment and password updating. The registration and the session key establishment phases are shown in Fig 2.

### 5.1 System initialization

The server *S* performs the following steps:

Step 1: Selects a random number $x\in Z_{p}$;

Step 2: Selects a private key *k* ∈ [1, *p* + 1] and computes *T*_*k*_(*x*)*modp* as its public key;

Step 3: Selects a chaotic map hash function *h*(), *S* maintains the secret key *k* and releases the parameters {*p*, *x*, *T*_*k*_(*x*)*modp*, *h*()}.

### 5.2 Registration

The registration phase of *A*/*B* as below:

Step 1: User *A*/*B* submits {*ID*_*A*_, *g*_*A*_ = *h*_1_(*pw*_*A*_, *r*_*A*_)}/{*ID*_*B*_, *g*_*B*_ = *h*_1_(*pw*_*B*_, *r*_*B*_)} to the server *S*, where *r*_*A*_ and *r*_*B*_ are the random numbers;

Step 2: Upon receiving the registration request, *S* computes *VPW*_*A*_ = *h*_1_(*ID*_*A*_, *k*) ⊕ *g*_*A*_/ *VPW*_*B*_ = *h*_1_(*ID*_*B*_, *k*) ⊕ *g*_*B*_. Next, *S* randomly chooses a secret key *r* for *A* and sends it to *A* via the private channel. Noth that *r* is kept securely by *A* and is different for each user *A*. Finally, *S* stores *k* ⊕ *r* and *VPW*_*A*_/*VPW*_*B*_ into its memory.

### 5.3 Session key establishment

After registering the server *S*, users *A* and *B* establish the session key with the help of *S* in the following manner:

Step 1: Using the stored shared secret key *r*, user *A* computes his own version of *C*_*A*_ = *E*_*K*_*AS*__(*ID*_*A*_, *ID*_*B*_, *T*_*a*_(*x*), *F*_*A*_) and sends them to *S*, where *K*_*AS*_ = *T*_*r*_(*T*_*k*_(*x*)), *F*_*A*_ = *h*(*ID*_*A*_, *ID*_*B*_, *T*_*a*_(*x*), *g*_*A*_) and *a* ∈ [*p* + 1] is a random number.

Step 2: Once receiving the message, *S* first derives *r* by computing *k* ⊕ *r* ⊕ *k* and derives {*ID*_*A*_, *ID*_*B*_, *T*_*a*_(*x*), *F*_*A*_} by decrypting *C*_*A*_ with computed symmetric key *K*_*AS*_ = *T*_*k*_(*T*_*r*_(*x*)). Next, *S* checks whether $h\left(ID_{A},ID_{B},T_{a}\left(x\right),g_{A}\right)\overset{?}{=}F_{A}$, where *g*_*A*_ = *VPW*_*A*_ ⊕ *h*(*ID*_*A*_, *k*). If the equation is true, *S* computes *C*_*B*_ = *E*_*g*_*B*__(*T*_*a*_(*x*), *F*_*B*_, *ID*_*A*_, *ID*_*B*_) and sends back it to user *B*, where *F*_*B*_ = *h*(*T*_*a*_(*x*), *ID*_*B*_).

Step 3: After receipt of the authentication message from *S*, user *B* first retrieves {*T*_*a*_(*x*), *ID*_*A*_, *ID*_*B*_, *F*_*B*_} by decrypting *C*_*B*_ and checks the validness of *F*_*B*_. If it is correct, *B* computes *P*_*B*_ = *E*_*g*_*B*__(*T*_*b*_(*x*), *H*_*B*_) and sends back an authentication message via an unsecure channel to *S* with the following values {*P*_*B*_}, where *H*_*B*_ = *h*(*ID*_*B*_, *T*_*b*_(*x*)) and *b* ∈ [1, *p* + 1] is a random number at *B* side.

Step 4: *S* decrypts *P*_*B*_ to get *T*_*b*_(*x*) and *H*_*B*_ using *g*_*B*_. After that, *S* examines whether $h\left(ID_{B},T_{b}\left(x\right)\right)\overset{?}{=}H_{B}$. If it is correct, *S* computes *Z*_*AS*_ = *h*(*ID*_*A*_, *ID*_*B*_, *T*_*b*_(*x*), *T*_*S*1_(*x*)), *R*_*AS*_ = *E*_*K*_*AS*__(*T*_*S*1_(*x*), *T*_*b*_(*x*), *ID*_*A*_, *Z*_*AS*_) and returns *R*_*AS*_ to *A*, where *S*1 is the random number and *K*_*AS*_ = *T*_*k*_(*T*_*r*_(*x*)) is a shared key between *A* and *S*. At the same time, *S* also computes *Z*_*BS*_ = *h*(*ID*_*A*_, *ID*_*B*_, *T*_*a*_(*x*), *T*_*S*2_(*x*)), *R*_*BS*_ = *E*_*K*_*BS*__(*T*_*S*2_(*x*), *T*_*a*_(*x*), *ID*_*B*_, *Z*_*BS*_) and returns *R*_*BS*_ to *B*, where *S*2 is the random number and *K*_*BS*_ = *T*_*k*_(*T*_*b*_(*x*)).

Step 5: When receiving the message from *S*, *A* checks whether $h\left(ID_{A},ID_{B},T_{b}\left(x\right),T_{S1}\left(x\right)\right)\overset{?}{=}Z_{AS}$ which is decrypted from *R*_*AS*_. If it holds, *A* computes the session key *SK* = *T*_*a*_(*T*_*b*_(*x*)) and *V*_*A*_ = *h*(*ID*_*A*_, *SK*), and then sends *V*_*A*_ to *B*. Similarly, *B* verifies the validity of *Z*_*BS*_ = *h*(*ID*_*A*_, *ID*_*B*_, *T*_*a*_(*x*), *T*_*S*2_(*x*)) which is derived from *R*_*BS*_. If it holds, *B* computes the session key *SK* = *T*_*b*_(*T*_*a*_(*x*)) and *V*_*B*_ = *h*(*ID*_*B*_, *SK*), and then sends *V*_*B*_ to *A*.

Step 6: Upon receiving the message from *B*, *A* verifies whether *h*(*ID*_*B*_, *SK*) is equal to the received *V*_*B*_. If the verification holds, *A* negotiates *SK* as the shared session key to encrypt the following messages. Otherwise, *A* aborts the session. At the same time, *B* checks the correctness of *V*_*B*_ = *h*(*ID*_*A*_, *SK*). Once the result is true, *B* agrees the session key *SK* with *A*.

### 5.4 Password update

When *A* intends to change his password after successful handshake between *A* and *S*, he can perform the following steps:

Step 1: *A* selects a new password $pw_{A}^{*}$ and computes $R_{A}=E_{T_{r}\left(x\right)}\left(ID_{A},h_{1}\left(pw_{A}^{*},r_{A}\right),h_{1}\left(pw_{A},r_{A}\right),Z_{AS}\right)$ and *Z*_*AS*_ = *h*(*ID*_*A*_, *T*_*S*1_(*x*), *K*_*AS*_) to *S*.

Step 2: *S* decrypts *R*_*A*_ to retrieve $\left\{ID_{A},h_{1}\left(pw_{A}^{*},r_{A}\right),h_{1}\left(pw_{A},r_{A}\right),Z_{AS}\right\}$ using the shared secret key *r* and verifies whether $h\left(ID_{A},T_{S1}\left(x\right),K_{AS}\right)\overset{?}{=}Z_{AS}$. If it is correct, *S* computes $VPW_{A}^{*}=h_{1}\left(pw_{A},r_{A}\right)⊕VPW_{A}⊕h_{1}\left(pw_{A}^{*},r_{A}\right)$. Next, *S* updates *VPW*_*A*_ with $VPW_{A}^{*}$.

If *B* plans to change his password into a new one after successful authentication process between *B* and *S*, he performs the following steps:

Step 1: *B* selects a new password $pw_{B}^{*}$ and computes $R_{B}=E_{K_{BS}}\left(ID_{B},h_{1}\left(pw_{B}^{*},r_{B}\right),h_{1}\left(pw_{B},r_{B}\right),Z_{BS}\right)$ and *Z*_*BS*_ = *h*(*ID*_*B*_, *T*_*S*2_(*x*), *K*_*BS*_) to *S*.

Step 2: *S* decrypts *R*_*B*_ to retrieve $\left\{ID_{B},h_{1}\left(pw_{B}^{*},r_{B}\right),h_{1}\left(pw_{B},r_{B}\right),Z_{BS}\right\}$ by the shared key *K*_*BS*_ and verifies whether $h\left(ID_{B},T_{S2}\left(x\right),K_{BS}\right)\overset{?}{=}Z_{BS}$. If it is correct, *S* computes $VPW_{B}^{*}=h_{1}\left(pw_{B},r_{B}\right)⊕VPW_{B}⊕h_{1}\left(pw_{B}^{*},r_{B}\right)$. Next, *S* updates *VPW*_*B*_ with $VPW_{B}^{*}$.

## 6 Security analysis of the proposed scheme

In this part, we first present a formal security analysis and then adopt the well-known formal tool for analyzing cryptographic protocol, i.e., BAN logic, to demonstrate the validness of the established session key between *A* and *B* in the help of the server *S*. After that, we conduct a security discussion for the proposed scheme according to the known kinds of security attributes. Next, we adopt the formal verification software to demonstrate our scheme is secure.

### 6.1 Formal security proof of the proposed scheme

Based on the one-way property of hash function [16] and ciphertext indistinguishability of symmetric cryptography algorithm [37], this part gives the formal analysis of the proposed scheme.

*Symmetric cryptography algorithm* Θ *assumption*: Denote the Θ advantage by $Adv_{P}^{\Theta }$. Θ is secure if $Adv_{P}^{\Theta }$ is negligible for any probabilistic, polynomial time adversary.

***Theorem 1*** Let Θ is secure. Assume that the one-way hash function *h*(⋅) behaves as a random oracle, then our proposed password-authentication key agreement defends against an adversary U for extracting the identity *ID*_*A*_ of the user *A*, and the session key *SK* between the user *A* and the user *B*.

*Reveal 1*: This oracle unconditionally outputs the cleartext *m* using symmetric cryptography algorithm Θ under the corresponding ciphertext *C* = *Enc*_*k*_(*m*).

*Reveal 2*: This oracle unconditionally outputs the input *x* using hash function under the corresponding hash value *y* = *h*(*x*).

***Proof.*** The adversary U executes the experiments $Exp1_{U,TPPPAKA}^{\Theta }$ (Table 1) and $Exp2_{U,TPPPAKA}^{Hash}$ (Table 2) for our three-party password-authentication key agreement. Suppose that the adversary U could get the identity *ID*_*A*_ of the user *A*, and the session key *SK* between the user *A* and the user *B*, which means U has an extremely high probability $Max_{U}Succ_{1}$ and $Max_{U}Succ_{2}$ to win the game within the running time *t*_*i*_ and the number of queries *q*_*i*_(*i* = 1, 2), where $Succ_{1}=|Pr\left(Exp1_{U,TPPPAKA}^{\Theta }=1\right)-1$ and $Succ_{2}=|Pr\left(Exp2_{U,TPPPAKA}^{Hash}=1\right)-1$. However, they are both computationally infeasible problems under the symmetric cryptography algorithm Θ assumption without the knowledge of the secret key *k* and non-invertibility of hash function, i.e., $Adv_{U,TPPPAKA}^{\Theta }\left(t_{1}\right)⩽\varepsilon_{1}$, $Adv_{U,TPPPAKA}^{hash}\left(t_{2}\right)⩽\varepsilon_{2}$, for any sufficiently small *ε*_*i*_ > 0(*i* = 1, 2). That is, $Max_{U}Succ_{1}⩽\varepsilon_{1}$ and $Max_{U}Succ_{2}⩽\varepsilon_{2}$ since both they depend on the advantage $Adv_{U,TPPPAKA}^{\Theta }$ and $Adv_{U,TPPPAKA}^{hash}$, respectively. As a result, no adversary U has the ability to derive the identity *ID*_*i*_ of the *A* and the session key *SK* between the user *A* and the user *B*.

**Table 1:** Algorithm 1.

1. Intercept the login message {*C*_*A*_}, *C*_*A*_ = *E*_*K*_*AS*__(*ID*_*A*_, *ID*_*B*_, *T*_*a*_(*x*), *F*_*A*_)
2. Call Reveal oracle 1. Let $\left(ID_{A}^{\prime },ID_{B}^{\prime },T_{a}{\left(x\right)}^{\prime },F_{A}^{\prime }\right)\leftarrow Reveal\left(C_{A}\right)$
3. Intercept the authenticated message {*C*_*B*_}, where *C*_*B*_ = *E*_*g*_*B*__(*ID*_*A*_, *ID*_*B*_, *T*_*a*_(*x*), *F*_*B*_)
4. Call Reveal oracle 1. Let $\left(ID_{A}^{\prime \prime },ID_{B}^{\prime \prime },T_{a}{\left(x\right)}^{\prime \prime },F_{B}^{\prime \prime }\right)\leftarrow Reveal\left(C_{B}\right)$
5. **If** (*T*_*a*_(*x*)′′ = *T*_*a*_(*x*)′) **then**
6. Accept $ID_{A}^{\prime }$ as the true identity of the user *A*
7. **return 1**
8. **else**
9. **return 0**
10. **end if**

**Table 2:** Algorithm 2.

1. Intercept the login message {*V*_*A*_}, where *V*_*A*_ = *h*(*ID*_*A*_, *SK*)
2. Call Reveal oracle 2. Let $\left(ID_{A}^{\prime },SK^{\prime }\right)\leftarrow Reveal\left(V_{A}\right)$
3. Intercept the authenticated message Intercept the login message {*V*_*A*_},
where *V*_*B*_ = *h*(*ID*_*B*_, *SK*)
4. Call Reveal oracle 1. Let $\left(ID_{A}^{\prime \prime },SK^{\prime \prime }\right)\leftarrow Reveal\left(V_{B}\right)$
5. **If** ($ID_{A}^{\prime }=ID_{A}^{\prime \prime }$) **then**
6. Accept *SK*′ as the correct session key between *A* and *B*
7. **return 1**
8. **else**
9. **return 0**
10. **end if**

### 6.2 Authentication proof based on BAN logic

BAN logic is an important formal mean and is widely applied for the security analysis of authentication schemes. Verification process for the protocol using BAN logic is mainly composed of four parts: ***Goals***, ***Idealisation***, ***Assumptions*** and ***Analysis***. Goals, as its name suggests, the objectives of the verification; Idealisation aims at formulating the protocol step in a way for each ciphertext communication; Assumptions state some essential information, such as, which principals have generated which fresh random numbers, what keys are originally shared between the principals, and which principals are trusted in special ways. Upon all the aforementioned basis, BAN logic analysis on the protocol step by step is a natural procedure. BAN logic defines some notations and rules to verify whether the mutual authentication is achieved between corresponds. We first introduce some common notations and rules related with our analysis in the following.

**Notations**

*P* ⊲ *X*: principal *P* sees a message containing *X*

*P*| ≡ *X*: *P* believes *X* is true

*P*| ∼ *X*: *P* is known to have sent a message including *X*

$P\overset{K}{↔}Q$: *P* and *Q* communicate with a shared key *K*

#*X*: formula *X* is fresh

*P* ⇒ *X*: *P* has jurisdiction over *X*

<*X*, *Y*>_*K*_: *X* and *Y* are encrypted with the key *K*

{*X*, *Y*}: *X* or *Y* is a part of the message {*X*, *Y*}

$\frac{Statement1,Statement2}{Statement3}$: a conjunction of *statements*1 and 2 can infer *statement*3

**Rules**

$\frac{A|\equiv A\overset{K}{↔}B,\;A⊲{\left\{X\right\}}_{K}}{A|\equiv |B∼X}$(Message-meaning rule): if *A* believes that the key *K* is shared with *B* and and receives a message containing *X* encrypted under *K*, then *A* believes that *B* once said *X*.

$\frac{A|\equiv \#X,\;A|\equiv B|∼X}{A|\equiv B|\equiv X}$(Nonce-verification rule): if *A* once said *X*, and *A* believes that *B* once said *X*, then *A* believes that *A* believes *X*.

$\frac{A|\equiv \#X}{A|\equiv \#(X,\;Y)}$(Fresh conjuncatenation rule): if *A* believes a component of a formula (*X*, *Y*) is fresh, then *A* believes the formula is fresh.

$\frac{A|\equiv B\Rightarrow X,\;A|\equiv B|\equiv X}{A|\equiv X}$(Jurisdiction rule): if *A* believes that *B* has controlled over *X*, and *A* believes that *B* believes *X*, then *A* trusts *B* on the truth of *X*.

(1) We establish the following ***goals*** which the session key agreement protocol should achieve:

$goal_{1}.\;A|\equiv A\overset{SK}{⟷}B$

$goal_{2}.\;A\left|\equiv B\right|\equiv A\overset{SK}{⟷}B$

*goal*_3_. *A*| ≡ *B*| ≡ *ID*_*B*_

$goal_{4}.\;B|\equiv A\overset{SK}{⟷}B$

*goal*_5_. *B*| ≡ *ID*_*A*_

*goal*_6_. *B*| ≡ *A*| ≡ *ID*_*A*_

$goal_{7}.\;B\left|\equiv A\right|\equiv A\overset{SK}{⟷}B$

(2) We ***idealize*** the communication messages of the proposed scheme as below:

*A* → *S*:

$C_{A}:<ID_{A},ID_{B},F_{A},T_{a}\left(x\right){>}_{A\overset{K_{AS}}{⟷}S}$,

$F_{A}:<ID_{A},ID_{B},T_{a}\left(x\right){>}_{A\overset{g_{A}}{⟷}S}$.

*S* → *A*:

$R_{AS}:<T_{S1}\left(x\right),T_{b}\left(x\right),Z_{AS},ID_{A}{>}_{A\overset{K_{AS}}{⟷}S}$,

*Z*_*AS*_: (*ID*_*A*_, *ID*_*B*_, *T*_*b*_(*x*), *T*_*S*1_(*x*)).

*S* → *B*:

$C_{B}:{\left\{T_{a}\left(x\right),ID_{A},ID_{B},F_{B}\right\}}_{A\overset{g_{B}}{⟷}S}$,

*F*_*B*_: *h*(*T*_*a*_(*x*), *ID*_*B*_),

$R_{BS}:<ID_{B},T_{S2}\left(x\right),T_{a}\left(x\right),Z_{BS}{>}_{B\overset{K_{BS}}{⟷}S}$,

*Z*_*BS*_: *h*(*ID*_*A*_, *ID*_*B*_, *T*_*a*_(*x*), *T*_*S*2_(*x*)).

*B* → *S*:

$P_{B}:<T_{b}\left(x\right),H_{B}{>}_{B\overset{g_{B}}{⟷}S}$,

*H*_*B*_: (*ID*_*B*_, *T*_*b*_(*x*)).

*A* → *B*:

$V_{A}:<ID_{A},SK{>}_{A\overset{SK}{⟷}B}$.

*B* → *A*:

$V_{B}:<ID_{B},SK{>}_{A\overset{SK}{⟷}B}$.

(3) We make some initial ***assumptions*** for the proposed scheme as follows:

*A*_1_. *A*| ≡ #*a*

*A*_2_. *B*| ≡ #*b*

*A*_3_. *B*| ≡ *ID*_*B*_

*A*_4_. *A*| ≡ *ID*_*A*_

$A_{5}.\;A|\equiv A\overset{K_{AS}}{⟷}S$

$A_{6}.\;S|\equiv A\overset{K_{AS}}{⟷}S$

*A*_7_. *A*| ≡ *ID*_*B*_

$A_{8}.\;A|\equiv A\overset{T_{r}\left(x\right)}{⟷}S$

$A_{9}.\;S|\equiv A\overset{T_{r}\left(x\right)}{⟷}S$

$A_{10}.\;A|\equiv A\overset{T_{aS1}\left(x\right)}{⟷}S$

$A_{11}.\;S|\equiv A\overset{T_{aS1}\left(x\right)}{⟷}S$

$A_{12}.\;B|\equiv B\overset{T_{bS2}\left(x\right)}{⟷}S$

$A_{13}.\;S|\equiv B\overset{T_{bS2}\left(x\right)}{⟷}S$

$A_{14}.\;B|\equiv B\overset{K_{BS}}{⟷}S$

$A_{15}.\;B|\equiv B\overset{g_{B}}{⟷}S$

Now, using the rules of the BAN logic, we ***demonstrate*** the proposed scheme can attain the intended goals based on the above descriptions:

According to the message *C*_*A*_, we derive:

*D*_1_. $S⊲<ID_{A},ID_{B},F_{A},T_{a}\left(x\right){>}_{A\overset{K_{AS}}{⟷}S}$

According to *A*_6_, *D*_1_ and message-meaning rule, we get:

*D*_2_. $\frac{S⊲<ID_{A},ID_{B},F_{A},T_{a}\left(x\right){>}_{A\overset{K_{AS}}{⟷}S},\;S|\equiv A\overset{K_{AS}}{⟷}S}{S|\equiv A|∼\{ID_{A},ID_{B},F_{A},T_{a}\left(x\right)\}}$

According to *R*_*AS*_, we obtain:

*D*_3_. $A⊲<T_{S1}\left(x\right),T_{b}\left(x\right),Z_{AS},ID_{A}{>}_{A\overset{K_{AS}}{⟷}S}$

According to *A*_5_, *D*_3_ and message-meaning rule, we get:

*D*_4_. $\frac{A⊲<T_{S1}\left(x\right),T_{b}\left(x\right),Z_{AS},ID_{A}{>}_{A\overset{K_{AS}}{⟷}S},A|\equiv A\overset{K_{AS}}{⟷}S}{A|\equiv S|∼\{T_{S1}\left(x\right),T_{b}\left(x\right),Z_{AS},ID_{A}\}}$

According to *D*_4_, *A*_4_ and fresh conjuncatenation rule, we obtain:

*D*_5_. $\frac{A|\equiv ID_{A},A\left|\equiv S\right|∼\left\{T_{S1}\left(x\right),T_{b}\left(x\right),Z_{AS},ID_{A}\right\}}{A|\equiv \{T_{b}\left(x\right),T_{S1}\left(x\right),Z_{AS}\}}$

According to *D*_5_, we immediately retrieve:

*D*_6_. $\frac{A|\equiv \{T_{S1}\left(x\right),T_{b}\left(x\right),Z_{AS}\}}{A|\equiv T_{S1}\left(x\right),A\left|\equiv T_{b}\left(x\right),A\right|\equiv Z_{AS}}$

According to *D*_6_, *SK* = *T*_*a*_(*T*_*b*_(*x*)) and *A*_1_, we also eventually achieve:

*goal*_1_. $\frac{A|\equiv T_{b}\left(x\right),SK=T_{a}\left(T_{b}\left(x\right)\right),A|\equiv \#a}{A|\equiv A\overset{SK}{⟷}B}$

According to the message *V*_*B*_, we gain:

*D*_7_. $A⊲{\left(ID_{B},SK\right)}_{A\overset{SK}{⟷}B}$

According to *D*_7_, *goal*_1_ and message-meaning rule, we get:

*D*_8_. $\frac{A⊲{\left(ID_{B},A\overset{SK}{⟷}B\right)}_{SK},A|\equiv A\overset{SK}{⟷}B}{A|\equiv B|∼\{ID_{B},A\overset{SK}{⟷}B\}}$

According to *goal*_1_, *D*_8_ and nonce-verification rule, we attain:

*goal*_2_. $\frac{A|\equiv A\overset{SK}{⟷}B,A\left|\equiv B\right|∼A\overset{SK}{⟷}B}{A|\equiv B|\equiv A\overset{SK}{⟷}B}$

According to *D*_8_, *A*_7_ and nonce-verification rule, we achieve:

*goal*_3_. $\frac{A|\equiv ID_{B},A\left|\equiv B\right|∼ID_{B}}{A|\equiv B|\equiv ID_{B}}$

According to the message *R*_*BS*_, we extract:

*D*_9_. $B⊲<ID_{B},T_{S2}\left(x\right),T_{a}\left(x\right),Z_{BS}{>}_{B\overset{K_{BS}}{⟷}S}$

According to *A*_14_, *D*_9_ and message-meaning rule, we collect:

*D*_10_. $\frac{B⊲<ID_{B},T_{S2}\left(x\right),T_{a}\left(x\right),Z_{BS}{>}_{B\overset{K_{BS}}{⟷}S},B|\equiv B\overset{K_{BS}}{⟷}S}{B|\equiv S|∼\{ID_{B},T_{S2}\left(x\right),T_{a}\left(x\right),Z_{BS}\}}$

According to *A*_3_, *D*_10_ and fresh conjuncatenation rule, we acquire:

*D*_11_. $\frac{B|\equiv ID_{B},B\left|\equiv S\right|∼\left\{ID_{B},T_{S2}\left(x\right),T_{a}\left(x\right),Z_{BS}\right\}}{B|\equiv \{T_{S2}\left(x\right),T_{a}\left(x\right),Z_{BS}}$

According to *D*_11_, we intuitively collect:

*D*_12_. $\frac{B|\equiv \{T_{S2}\left(x\right),T_{a}\left(x\right),Z_{BS}\}}{B|\equiv T_{S2}\left(x\right),B\left|\equiv T_{a}\left(x\right),B\right|\equiv Z_{BS}}$

According to *A*_2_, *D*_12_ and *SK* = *T*_*b*_(*T*_*a*_(*x*)), we naturally receive:

*goal*_4_. $\frac{B|\equiv T_{a}\left(x\right),SK=T_{b}\left(T_{a}\left(x\right)\right),B|\equiv \#b}{B|\equiv A\overset{SK}{⟷}B}$

According to the message *C*_*B*_, we obtain:

*D*_13_. $B⊲{\left\{T_{a}\left(x\right),ID_{A},ID_{B},F_{B}\right\}}_{A\overset{g_{B}}{⟷}S}$

According to *A*_15_, *D*_13_ and message-meaning rule, we attain:

*D*_14_. $\frac{B⊲{\left\{T_{a}\left(x\right),ID_{A},ID_{B},F_{B}\right\}}_{A\overset{g_{B}}{⟷}S},B|\equiv B\overset{g_{B}}{⟷}S}{B|\equiv S|∼\{T_{a}\left(x\right),ID_{A},ID_{B},F_{B}\}}$

According to *A*_3_, *D*_14_ and fresh conjuncatenation rule, we derive:

*goal*_5_. $\frac{B|\equiv ID_{B},B\left|\equiv S\right|∼\left\{T_{a}\left(x\right),ID_{A},ID_{B},F_{B}\right\}}{B|\equiv \{ID_{A}\}}$

According to *V*_*A*_, we collect:

*D*_15_. $B⊲{\left(ID_{A},SK\right)}_{A\overset{SK}{⟷}B}$

According to *A*_15_, *goal*_4_ and message-meaning rule, we attain:

*D*_16_. $\frac{B⊲{\left(ID_{A},A\overset{SK}{⟷}B\right)}_{SK},B|\equiv A\overset{SK}{⟷}B}{B|\equiv A|∼\{A\overset{SK}{⟷}B,ID_{A}\}}$

According to *goal*_5_, *D*_16_ and nonce-verification rule, we get:

*goal*_6_. $\frac{B|\equiv ID_{A},B\left|\equiv A\right|∼ID_{A}}{B|\equiv A|\equiv ID_{A}}$

According to *goal*_4_, *goal*_5_ and nonce-verification rule, we get:

*goal*_7_. $\frac{B|\equiv A|∼A\overset{SK}{⟷}B,B|\equiv A\overset{SK}{⟷}B}{B|\equiv A|\equiv A\overset{SK}{⟷}B}$

### 6.3 Informal security analysis

In this part, we demonstrate the strong ability of the proposed scheme. Specifically, we will show that the proposed scheme is secure against the loopholes which found in the scheme of Xie et al. Besides, the proposed scheme also provide other common security features. To facilitate the discussion, we also adopt the attack model proposed by Xu et al. [35], that is, an adversary can completely monitor the open communication channel, thus inserting, deleting, and modifying any messages among correspondents.

#### 6.3.1 User anonymity

We employ symmetric cryptography to safeguard user identity. Specifically, the identities {*ID*_*A*_, *ID*_*B*_} are contained only in *C*_*A*_, *R*_*AS*_ or *C*_*B*_, *G*_*B*_ and *R*_*BS*_ in the form of ciphtertext, where *C*_*A*_ = *E*_*K*_*AS*__(*ID*_*A*_, *ID*_*B*_, *F*_*A*_), *R*_*AS*_ = *E*_*K*_*AS*__(*T*_*S*1_, *T*_*b*_(*x*), *Z*_*AS*_), *Z*_*AS*_ = *h*(*ID*_*A*_, *ID*_*B*_, *T*_*b*_(*x*), *T*_*S*_1__(*T*_*a*_(*x*))), *C*_*B*_ = *E*_*g*_*B*__(*T*_*a*_(*x*), *h*(*T*_*a*_(*x*), *T*_*g*_*B*__(*ID*_*B*_)), *G*_*B*_ = *E*_*K*_*BS*__(*ID*_*B*_, *H*_*B*_), *R*_*BS*_ = *E*_*K*_*BS*__(*T*_*S*2_, *T*_*a*_(*x*), *Z*_*BS*_), *Z*_*BS*_ = *h*(*ID*_*A*_, *ID*_*B*_, *T*_*a*_(*x*)), *K*_*AS*_ = *T*_*a*_(*T*_*k*_(*x*)), *g*_*A*(*B*)_ = *h*_1_(*pw*_*A*(*B*)_, *r*_*A*(*B*)_). From the above we can see that both the identities of *A* and *B* are protected by the server’s public key, chaotic-maps, hash function and symmetric cryptographic operations. Besides, used parameters include secret keys and random numbers are not exposed in the public channel. For example, suppose an adversary U eavesdrops the message *C*_*A*_ and he plans to derive the identity of *A*. He first needs to know *K*_*AS*_ = *T*_*a*_(*T*_*k*_(*x*)). To obtain *T*_*a*_(*x*) from intercepted *H*_*A*_ = *T*_*a*_(*x*) ⊕ *T*_*r*_(*x*), the shared secret key *r* is needed. In general, it is hard to derive from the transmitted messages. Our proposed scheme is therefore secure from trace attack.

#### 6.3.2 Avoidance of insider attack

In the registration phase of our proposed scheme, *A* and *B* send *g*_*A*_ = *h*_1_(*pw*_*A*_, *r*_*A*_) or *g*_*B*_ = *h*_1_(*pw*_*B*_, *r*_*B*_) to the server *S*, respectively. When *S* receiving the registration request, he cannot retrieve the cleartext password *pw*_*A*_ or *pw*_*B*_ owing to the unawareness of the random numbers *r*_*A*_ and *r*_*B*_. Therefore, the proposed scheme can protect against the insider attack.

#### 6.3.3 Avoidance of off-line password guessing attack

U intercepts all the communicated messages {*C*_*A*_, *H*_*A*_, *C*_*B*_, *P*_*B*_, *G*_*B*_, *R*_*AS*_, *R*_*BS*_}, he still cannot derive password of user *B*. Assume that U steals the stored information {*VPW*_*A*_} or {*VPW*_*B*_}, where *VPW*_*A*(*B*)_ = *h*_1_(*pw*_*A*(*B*)_, *r*_*A*(*B*)_) ⊕ *h*_1_(*ID*_*A*(*B*)_, *k*). Even if the secret key *k* of *S* is compromised, U also requires the random number *r*_*A*(*B*)_. In addition, the identity of *A* or *B* is also needed. This point has been ensured by user anonymity. This means the off-line password guessing attack is not able to come true in our scheme.

#### 6.3.4 Avoidance of user impersonation attack

By virtue of being discussed in the previous subsection, U is not possible to guess the correct password, let alone masquerade as a legal user to cheat the services provided by the server *S*. Once U fabricates the password and sends the forged message {*C*_*A*_} or {*P*_*B*_} to the server *S*. After receiving the message, *S* will decrypt *C*_*A*_ by using its own private key *k*. It is clear that *S* will detect the attack from user by checking the correctness of *F*_*A*_ or *H*_*B*_ by using its own computed values *g*_*A*_ = *h*_1_(*pw*_*A*_, *r*_*A*_) = *VPW*_*A*_ ⊕ *h*_1_(*ID*_*A*_, *k*) or *g*_*B*_ = *h*_1_(*pw*_*B*_, *r*_*B*_) = *VPW*_*B*_ ⊕ *h*_1_(*ID*_*B*_, *k*). Therefore, U is also impossible to launch the user impersonation attack.

#### 6.3.5 Avoidance of man-in-the-middle attack

Assume that U intercepts the login message {*C*_*A*_ = *E*_*K*_*AS*__(*ID*_*A*_, *ID*_*B*_, *T*_*a*_(*x*), *F*_*A*_)} and attempts to modify it. However, he has no way to know the shared symmetric key *K*_*AS*_ between *A* and *S*. Without the important key, he is not possible to decrypt it. Similarly, if U eavesdrops the message *C*_*B*_ = *E*_*g*_*B*__(*T*_*a*_(*x*), *F*_*B*_, *ID*_*A*_, *ID*_*B*_) and plans to forge it. He also face an embarrassed reality without knowledge of the shared symmetric key *g*_*B*_. Therefore, the proposed scheme protects against the man-in-the middle attack. This point will be verified by the simulation result later.

#### 6.3.6 The session key perfect forward secrecy

The session key *SK* = *T*_*a*_(*T*_*b*_(*x*)), where *T*_*a*_(*x*) and *T*_*b*_(*x*) are not directly transmitted in the public channel. On the one side, *T*_*a*_(*x*) and *T*_*b*_(*x*) are encrypted with the symmetric cryptographic technology or the Chebyshev polynomials, where the symmetric key is *g*_*B*_ and chaotic map is *T*_*r*_(*x*). The security of symmetric key has been demonstrated in the previous subsection. On the other side, assume that U has the secret key of *S* and the stored information {*VPW*_*A*_} or {*VPW*_*B*_}. In this case, it is an impossible task for U to attempt to derive *g*_*A*_ or *g*_*B*_ due to the unknown of the identity *A* or *B*. In order to know the identity, which goes back to this discussion about user anonymity. Therefore, the proposed scheme is able to provide the session key perfect forward secrecy.

#### 6.3.7 Mutual authentication

*A* sent the message {*C*_*A*_, *H*_*A*_} to *S*, where *C*_*A*_ = *E*_*K*_*AS*__(*ID*_*A*_, *ID*_*B*_, *F*_*A*_), *F*_*A*_ = *h*(*ID*_*A*_, *ID*_*B*_, *T*_*a*_(*x*), *g*_*A*_) and *H*_*A*_ = *T*_*a*_(*x*) ⊕ *T*_*r*_(*x*). Upon receiving the message, *S* derives *T*_*a*_(*x*) using the shared secret key *r* and then decrypts *C*_*A*_ to get {*ID*_*A*_, *ID*_*B*_, *F*_*A*_} using its private key *k*. Next, *S* computes *h*(*ID*_*A*_, *ID*_*B*_, *T*_*a*_(*x*), *VPW*_*A*_ ⊕ *h*_1_(*ID*_*A*_, *k*)) and checks whether it is equal to the decrypted from *C*_*A*_. If it is correct, *A* is authenticated. The validness of *F*_*B*_ which is decrypted from *C*_*B*_ to verify the legitimacy of *S*. And the correctness of *H*_*B*_ which is decrypted from *G*_*B*_ to validate the legalization of *B*. Similarly, *A* authenticates *S* by checking the verification of *Z*_*AS*_ decrypted from *R*_*AS*_. Finally, the authentication between *A* and *B* are gone through the correctness of *V*_*A*_ and *V*_*B*_.

### 6.4 Formal validation of the proposed scheme using AVISPA software

**Fig 3:**
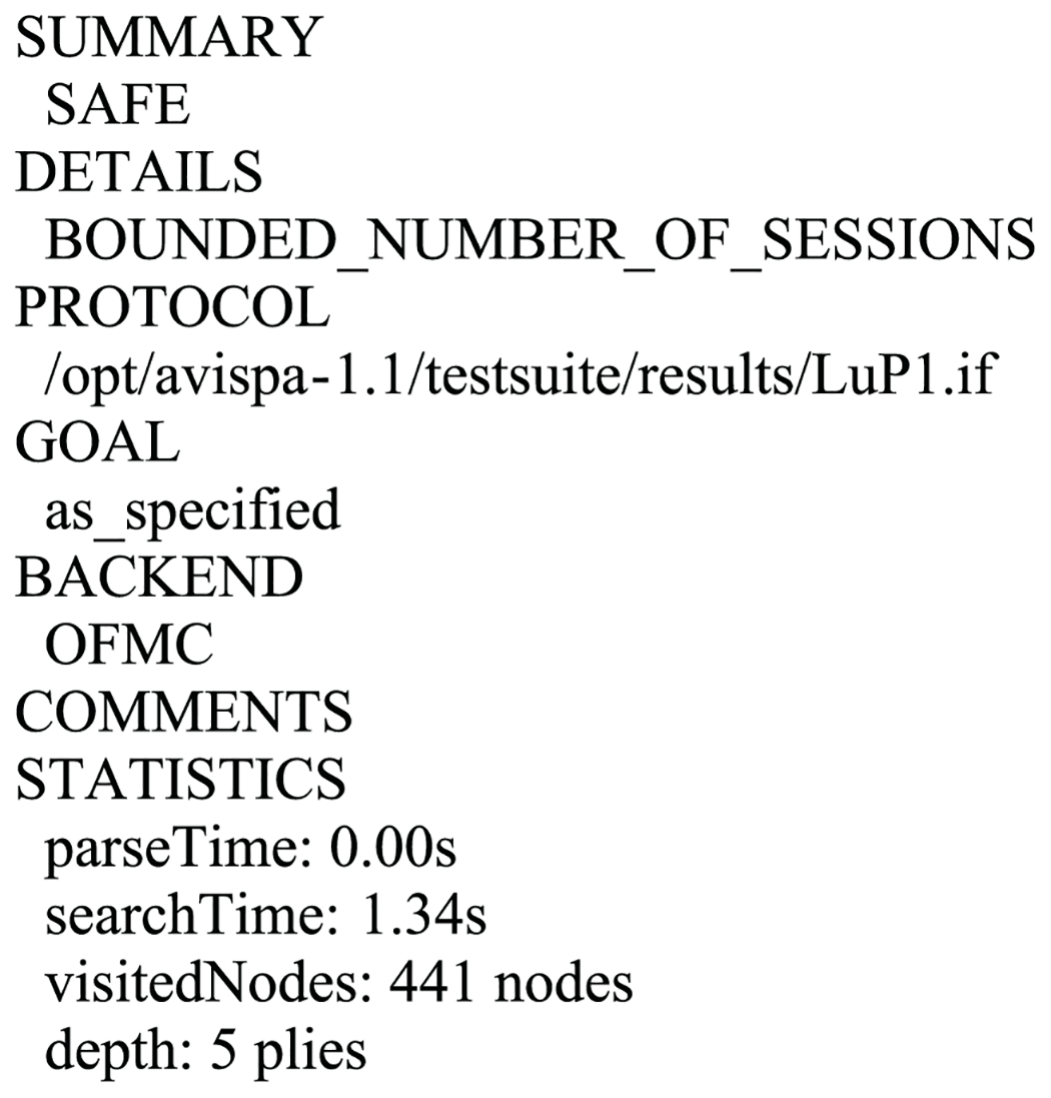
Simulation result for the OFMC.

**Fig 4:**
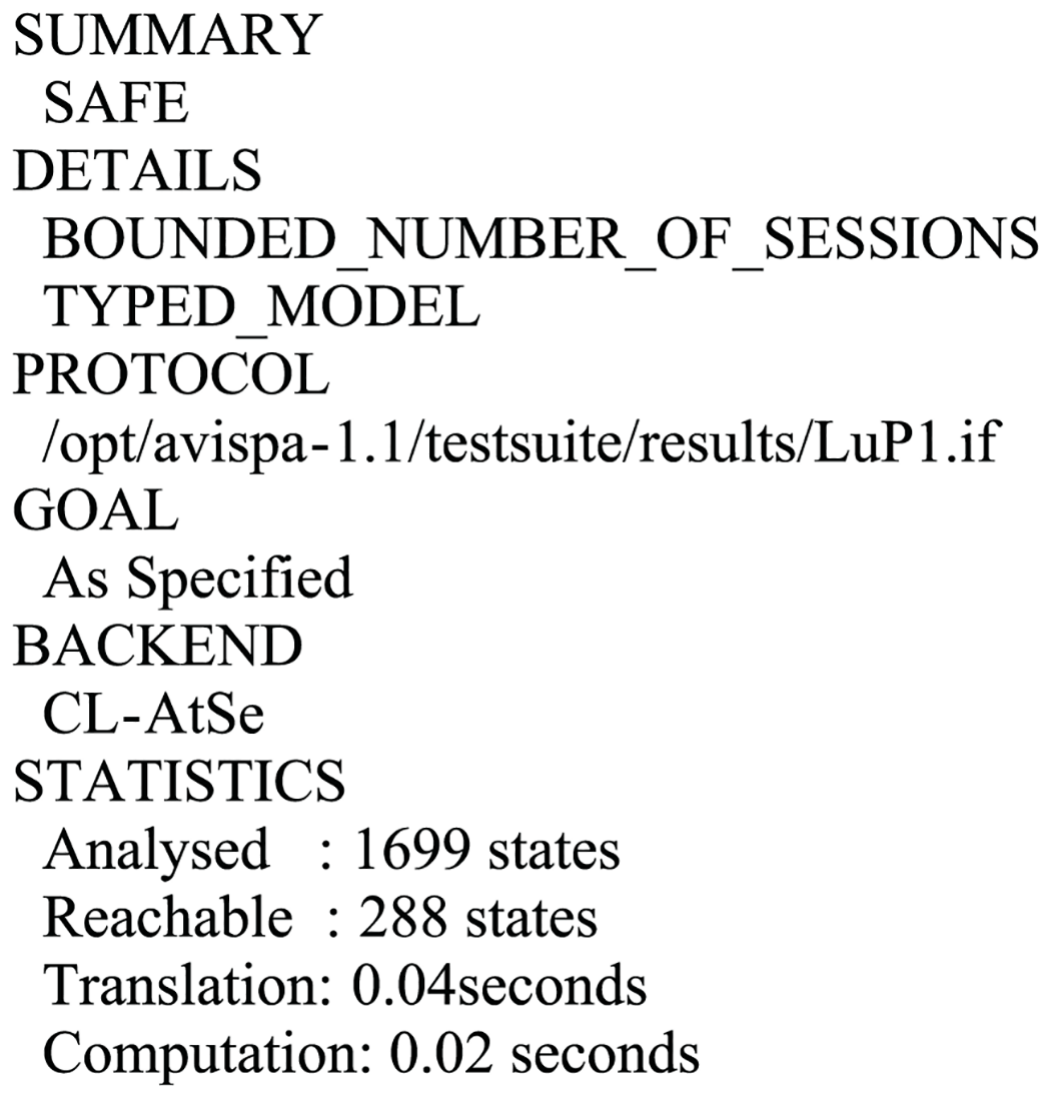
Simulation result for the CL-AtSe.

In this part, we simulate the proposed scheme using the commonly used AVISPA (Automated Validation of Internet Security Protocols and Applications) toolkit [30–31] to validate the passive and active attacks including man-in-the-middle and replay attacks that has been withstand. AVISPA integrates four backends: (i)OFMC; (ii)CL-AtSe; (iii)SATMC; (iv)TA4SP for the analysis of security schemes and implements in the role based HLPSL (High Level Protocol Specification Language). After execution through the OFMC and CL-AtSe backends, the results (Figs 3–4) clearly verify that the proposed scheme is secure under the Dolev-Yao model. The specifications for the roles for *U*_*A*_(S1 Fig), *U*_*B*_(S2 Fig), *S*(S3 Fig), the Session(S4 Fig) and the Environment(S5 Fig) in HLPSL are provided in Supporting Information.

## 7 Performance comparisons

In this section, we evaluate the performance of our proposed scheme and make comparisons with the recent chaotic-maps based schemes [28, 2, 4, 9]. The following types of computation costs will be used to evaluate the feasibility of the attack in terms of its computational complexity.

*T*_*cp*_: time for computing Chebyshev polynomial;

*T*_*h*_: time for computing hash function;

*T*_*S*_: time for performing symmetric cryptography;

*T*_*pm*_: time for computing point multiplication;

*T*_*m*_: time for performing MAC generation/verification.

Table 3 shows the computation overhead comparisons of our proposed scheme and some recent three-party schemes. We mainly address on the consumptions of authentication and session key agreement due to these are the principal parts of an authentication scheme and should be performed for each session. In Table 3, it is obvious that our improvements need a sight higher computational cost than Xie et al.’s scheme while consuming less than others, where the time for performing a point multiplication is much more expensive than the lightweight cryptographic operations, and a symmetric encryption/decryption operation is almost as many costs as a hash function [34]. However, it is worth an additional chaotic-maps and symmetric cryptographic operations to achieve strong security and better functionality attributes compared with Xie et al.’s scheme.

**Table 3:** Performance comparison.

	Ours	Xie et al. [28]	Chou et al. [2]	He-Wang [4]	Nam et al. [9]
User	3*T*_*cp*_ + 4*T*_*h*_ + 4*T*_*h*_	3*T*_*cp*_ + 3*T*_*h*_	3*T*_*pm*_ + 2*T*_*h*_	3*T*_*pm*_ + 7*T*_*h*_	3*T*_*pm*_ + 1*T*_*S*_ + 4*T*_*h*_ + 1*T*_*m*_
Second party	2*T*_*cp*_ + 3*T*_*S*_ + 5*T*_*h*_	3*T*_*cp*_ + 3*T*_*h*_	3*T*_*pm*_ + 2*T*_*h*_	2*T*_*pm*_ + 5*T*_*h*_	1*T*_*m*_ + 1*T*_*S*_ + 1*T*_*h*_
Third patry	5*T*_*cp*_ + 5*T*_*S*_ + 7*T*_*h*_	4*T*_*cp*_ + 6*T*_*h*_	3*T*_*pm*_ + 8*T*_*h*_	2*T*_*pm*_ + 9*T*_*h*_	1*T*_*m*_ + 1*T*_*S*_ + 2*T*_*h*_
Communication rounds	6	5	6	6	4

Table 4 lists the security comparisons among our proposed scheme and some recent three-party schemes. It demonstrates that our scheme has many excellent features and is more secure than other recent three-party schemes.

**Table 4:** Security properties comparison.

	Ours	Xie et al. [28]	Chou et al. [2]	He-Wang [4]	Nam et al. [9]
Session key perfect forward secrecy	Yes	No	Yes	Yes	Yes
Mutual authentication	Yes	Yes	Yes	Yes	Yes
User anonymity	Yes	No	No	Yes	Yes
Insider attack	Yes	Yes	-	Yes	No
Off-line password guessing attack	Yes	No	-	Yes	No
Impersonation attack	Yes	No	No	No	No

## 8 Conclusion and future work

This paper discussed the security of the recent scheme proposed by Xie et al. We showed that the recent scheme had several security pitfalls. Besides, we found that it was insecure only using hash function. To mend all the identified weaknesses, we then presented an enhancement which utilized asymmetric cryptography to conceal the user’s identity. We demonstrated that the improvements not only was immune to the loopholes found in Xie et al.’s scheme but also was secure other common attacks. We also performed the BAN logic test and confirmed the mutual authentication is achieved in our scheme. The formal security analysis also shows our scheme supports more security properties. The performance comparison between the recent schemes and the proposed scheme showed our improvements was more secure than other schemes. Actually, it is not negligible that based on chaotic maps has inevitable restrictions in some applications and an ID-based solution is a better one. Therefore, our near future work is to address to design a robust ID-based authenticated key agreement scheme.

## Supporting Information

S1 Fig Role specification of *U*_*A*_. (EPS)

S2 Fig Role specification of *U*_*B*_. (EPS)

S3 Fig Role specification of *S*. (EPS)

S4 Fig Role specification of the Session. (EPS)

S5 Fig Role specification of the Environment. (EPS)
